# Tuning the hierarchical pore structure of graphene oxide through dual thermal activation for high-performance supercapacitor

**DOI:** 10.1038/s41598-021-81759-7

**Published:** 2021-01-22

**Authors:** Jeongpil Kim, Jeong-Hyun Eum, Junhyeok Kang, Ohchan Kwon, Hansung Kim, Dae Woo Kim

**Affiliations:** Department of Chemical and Biomolecular Engineering, Yonsei University, Yonsei-ro 50, Seodaemun-gu, Seoul, 120-749 Republic of Korea

**Keywords:** Energy science and technology, Materials science, Nanoscience and technology

## Abstract

Herein, we introduce a simple method to prepare hierarchical graphene with a tunable pore structure by activating graphene oxide (GO) with a two-step thermal annealing process. First, GO was treated at 600 °C by rapid thermal annealing in air, followed by subsequent thermal annealing in N_2_. The prepared graphene powder comprised abundant slit nanopores and micropores, showing a large specific surface area of 653.2 m^2^/g with a microporous surface area of 367.2 m^2^/g under optimized conditions. The pore structure was easily tunable by controlling the oxidation degree of GO and by the second annealing process. When the graphene powder was used as the supercapacitor electrode, a specific capacitance of 372.1 F/g was achieved at 0.5 A/g in 1 M H_2_SO_4_ electrolyte, which is a significantly enhanced value compared to that obtained using activated carbon and commercial reduced GO. The performance of the supercapacitor was highly stable, showing 103.8% retention of specific capacitance after 10,000 cycles at 10 A/g. The influence of pore structure on the supercapacitor performance was systematically investigated by varying the ratio of micro- and external surface areas of graphene.

## Introduction

Supercapacitors are efficient energy-storage devices owing to their advantages, including high power density, fast charge/discharge rates, stable operation over a wide temperature range, and long cycle life^1–3^. To enhance their performance, the electrode materials are required to have a high specific surface area and suitable electrical conductivity; several carbon materials, such as activated carbon, carbon nanotubes, and graphene, have been widely utilized to meet these requirements^4–10^. In particular, hierarchical graphene comprising both micro- and mesopores is desirable for fabricating high-performance supercapacitors, because the micropores provide a high specific surface area for ion adsorption and the mesopores facilitate ion transport^11, 12^. Moreover, graphene is highly conductive, and thus, can efficiently collect electrons on the metal electrode surface^13, 14^.

Several methods have been suggested to prepare nanoporous and hierarchical graphene structures, such as self-assembly^15–17^, template approach^18, 19^, chemical vapor deposition (CVD)^20–25^, and chemical/thermal activation^26–30^. Graphene oxide (GO) can be self-assembled in the form of a macroporous scaffold in its concentrated solution^15^ or during a hydrothermal treatment^16^, and the graphene scaffold can be used as an electrode after reduction^17^. The template approach uses a sacrificial nanomaterial, such as polystyrene nanoparticles, as a template, which can be removed by a chemical or thermal etching process, leaving a graphene scaffold^18, 19^. CVD deposits a graphene layer on macroporous metal catalysts, including a Ni/Cu mesh, a metal nanowire, and metal nanoparticles, by treating the hydrocarbon and hydrogen gas at high temperatures^20–25^, and the metal catalyst is etched away using an acidic solution. Chemical activation is effective for preparing porous graphene in the form of bulk powder, which is generally conducted at 450–900 °C in the presence of activation agents such as KOH, H_3_PO_4_, and ZnCl_2_ to facilitate the transformation of solid carbon of graphene into CO_2_ and CO gas, leaving defective nanopores^26–30^. Although the above methods are effective for preparing porous graphene, the preparation processes are laborious because additional steps are required to remove the added chemicals or templates and the pore structure can be hardly controlled in the microporous dimension^31^. Therefore, a simple and scalable preparation method for graphene with a tunable pore structure is required to accelerate the commercialization of high-performance supercapacitors using graphene-based materials.

In this study, we developed a simple method to tune the hierarchical and porous structure of graphene materials through thermal annealing of GO without additional processes or additives. It was demonstrated that the micro- and mesopores of graphene can be precisely tuned by controlling the oxidation degree of GO, which was used as a starting material for thermal activation. The pore structure, particularly for micropores, was further modified by the second annealing treatment of the as-prepared nanoporous graphene in an inert environment. In addition, the influence of the hierarchical pore structure of graphene on the electrochemical performance of supercapacitors was systematically investigated.

## Experimental

### Synthesis of GO with controlled oxidation degree

Graphite (particle size 20 μm, 99% pure) was purchased from Sigma Aldrich and used as-received without purification. It was oxidized using a modified Hummer’s method^32, 33^. The ratio of graphite and KMnO_4_ was fixed at 3.5 for all GO preparation procedures. First, 2 g of graphite powder was added to 150 mL of sulfuric acid and stirred to form a homogeneous graphite solution. Then, potassium permanganate (KMnO_4_, 7 g) was gradually added to the graphite solution while stirring in an ice bath to cool the solution. After stirring for the desired time in a water bath at 35 °C, the solution was placed in an ice bath and 200 mL of deionized (DI) water and 100 mL of hydrogen peroxide (H_2_O_2_) were slowly added in sequence. The stirring time was varied from 30 min to 10 h to observe the influence of oxidation time on the pore structure of reduced GO (rGO) after rapid thermal annealing. Next, vacuum filtration was conducted to filter the resulting product with a cellulose filter and washed several times with DI water to remove the remaining chemicals. The filtered GO cake was dispersed in DI water and frozen for 48 h in a refrigerator, and then freeze-dried to obtain sponge-like GO powder.

### Pore activation of rGO

GO powder (250 mg) was loaded in a quartz tube, which was spontaneously placed in a 650 °C furnace for 3 min under ambient conditions to reduce and expand the powder by rapid thermal annealing. This process is explosive due to the spontaneous gas emission by the decomposed oxygen-containing groups of GO, and thus, special care needs to be taken while handling large quantities of the powder. Long thermal annealing is not recommended here because the carbons of the graphene flakes can rapidly decompose in air at high temperatures^34^. After rapid thermal annealing, the quartz tube was removed from the furnace and cooled down to room temperature naturally. After thermal annealing, the brown color of GO turned black. The resulting product, activated reduced graphene oxide (ArGO), was collected and stored as a powder. To further control the pore structure of the powder by increasing the numbers of micropores and reducing the number of oxygen groups, 200 mg of ArGO powder was loaded onto a crucible and placed in a furnace under N_2_ conditions at 600 °C for 2 h, resulting in thermally treated ArGO (TArGO) powder. Because of the inert environment with N_2_ gas, the decomposition of the graphene powder was highly suppressed and the electrical conductivity of graphene was enhanced.

### Characterization

The morphology of the obtained graphene product was observed using scanning electron microscopy (SEM, JSM-6701F, JEOL Ltd), and X-ray diffraction (XRD) measurements were performed using an Ultima IV (Rigaku) instrument with a wavelength of 1.54 Å. Raman spectra were acquired using a Labram Aramis (Horriba Jovin Yvon) at an excitation wavelength of 532 nm. X-ray photoelectron spectroscopy (XPS) was performed using a K-alpha (Thermo U.K.) with a Cu(Kα) beam source (wavelength 1.5406 Å), and Fourier transform-infrared spectra were obtained using a Cary670 (Agilent) spectrometer. N_2_ adsorption–desorption isotherms were measured using a BELSORP-mini II (BEL Japan) and the Barrett–Hoyner–Halenda (BJH) method was used to calculate the pore size distribution. The electrical conductivity of the carbon materials was calculated from the sheet resistance of the graphene pellet, which was measured using the four-probe method with a Keithley 2635A SYSTEM SourceMeter. Graphene pellets with a thickness of 100 µm were prepared by pressing the graphene powder at 25 °C with a pressure of 2.6 ton/cm^2^. The sheet resistance of the graphene pellets was converted to electrical conductivity, as follows:

1 $$\sigma =\frac{1}{Rs T}$$

Here σ is the electrical conductivity, Rs is the sheet resistance, and T is the thickness of the graphene pellets.

### Electrochemical characterization

The electrochemical performances of rGO-V20 (commercial rGO purchased from Standard Graphene), ArGO, and TArGO were measured in three-electrode configurations using Biologic VSP as an electrochemical station. For fabricating the working electrode, a slurry was prepared by mixing 2.83 mg of graphene powder with 50 μL of 5 wt% Nafion binder in isopropyl alcohol (1.5 mL) and DI water (0.5 mL). The slurry was dropped onto a glassy carbon electrode and dried in a nitrogen chamber. A saturated calomel electrode was used as the reference electrode, while a platinum wire was used as the counter electrode; a 1 M H_2_SO_4_ solution was used as the electrolyte. The specific capacitance (C_sp_) of the electrode was measured by galvanostatic charge and discharge (GCD) using the following equation:

2 $${C}_{sp}= \frac{I \Delta t}{m \Delta V}$$

Here I is the current, Δt is the discharging time, m is the mass of the active material, and ΔV is the potential window.

## Results and discussion

**Figure 1:**
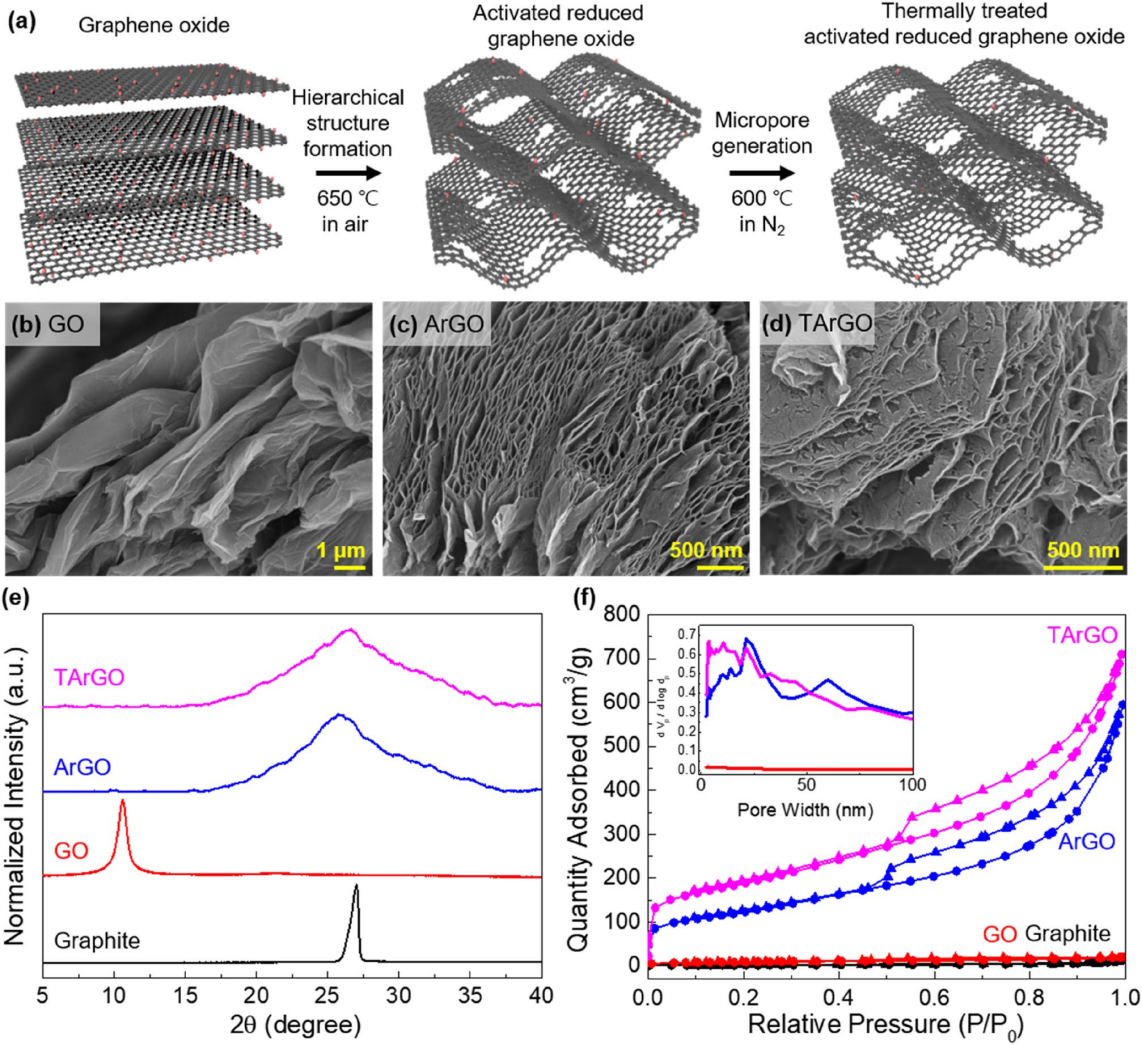
(**a**) Schematic for the preparation of thermally treated activated reduced graphene oxide (TArGO) powder. (**b**)**–**(**d**) Scanning electron microscopy images of GO and ArGO after the thermal treatment of ArGO particles. (**e**) X-ray diffraction patterns of graphite, GO, ArGO, and TArGO. (**f**) N_2_ adsorption–desorption isotherms of graphite, GO, ArGO, and TArGO at 77 K. The inset shows the pore size distributions of GO, ArGO, and TArGO calculated using the BJH method.

The procedure for the preparation of hierarchically porous graphene is illustrated in Fig. 1a. Briefly, GO powder was quickly placed in a thermal furnace at 650 °C in air. At high temperatures, the oxygen-containing groups spontaneously decompose into CO_2_/CO gas, which expands the graphene sheet to form a hierarchical structure with abundant slit nanopores (middle image of Fig. 1a)^32, 35^. Simultaneously, nanopores can be generated on the basal plane of graphene, because the oxygen-containing groups are detached. After cooling the ArGO, the powder was further treated at 600 °C under N_2_ to increase the number of micropores by further reducing the remaining oxygen-containing groups (right image of Fig. 1a). The powder obtained after the second thermal annealing is referred to as TArGO. The separation of the first and second thermal annealing is critical for increasing the graphene powder yield because graphene can be easily decomposed at high temperatures in air through the reaction between carbon and oxygen^36^. In addition, high-temperature annealing in inert gas is helpful for controlling the pore structure of rGO, because of the slow etching of carbon atoms under inert conditions^37^. Because the approach does not use any additives or chemical, the as-prepared graphene powder can be directly used for fabricating supercapacitor electrodes without any purification process.

Figure 1b–d present the SEM images of GO, ArGO, and TArGO particles, respectively. The SEM image in Fig. 1b displays GO particles composed of stacked GO nanosheets formed within an oxidation time of 30 min. The oxidation degree of the GO powder can be adjusted by controlling the oxidation time of the GO synthesis (Table S1; Fig. S1); an oxidation time of 30 min is primarily adopted in the description below, unless otherwise noted. After the first annealing in air, the ArGO particles became hierarchical and were composed of expanded graphene sheets with abundant slit pores with a gap size below 60 nm (Fig. 1c). Apparently, the effect of oxidation time is not significant for the slit-pore generation, showing a successful formation of the hierarchical structure of ArGO regardless of the oxidation degree (Fig. S2), while its effects on the formation of micropores are critical, as discussed later. Figure 1d shows that TArGO preserved the hierarchical structure of ArGO after the second thermal annealing, while more rough and defective structures were observed on the basal plane of graphene. This morphology variation is attributable to the decomposition of oxygen-containing groups and the etching of carbon atoms during the second thermal annealing, particularly with the unsaturated carbons located at the edge and defects of graphene^37^.

An XRD analysis was conducted to understand the interlayer structures of graphite, GO, ArGO, and TArGO shown in Fig. 1e. After thermal annealing, the d-spacing of ArGO (3.4 Å) decreased over that of GO powder (8.3 Å) because most oxygen-containing groups were removed by the high-temperature treatment. However, the diffraction peak of ArGO was broader than that of graphite because ArGO was mainly composed of amorphous sp^3^ carbons, hindering the stacking of graphene sheets^32, 35^, and a broad diffraction peak was observed regardless of the oxidation degree of GO (Fig. S3a). The d-spacing of TArGO slightly decreased to 3.3 Å because the residual oxygen-containing groups were further removed by the second thermal annealing. However, a sharp diffraction peak, such as the (002) pattern of graphite, was not observed even after the second thermal annealing at 600 °C for 2 h under inert conditions, because an extremely high temperature above 2300 °C is required to graphitize amorphous carbons in the absence of catalysts^38, 39^.

The N_2_ adsorption–desorption isotherms of graphite, GO, ArGO, and TArGO were evaluated to confirm the existence of micropores and mesopores in the graphene samples (Fig. 1f). ArGO and TArGO exhibited significant adsorption of N_2_ below the relative pressure (P/P_0_) of 0.1, indicating the presence of micropores, while the N_2_ adsorption of GO was negligible due to the stacked structure and the absence of micropores. In addition, ArGO and TArGO showed type-IV adsorption isotherms in the relative pressure range of 0.4–1.0, indicating the presence of slit-like mesopores, as observed in the SEM images in Fig. 1c,d. The pore size distribution derived from the N_2_ adsorption isotherm using the BJH model is also displayed in Fig. 1f, confirming the presence of micro- and mesopores in the ArGO and TArGO samples, while peaks were not observed in the GO sample.

The external pore volume and surface area were obtained by subtracting the micropore volume and micropore surface area, respectively, from the total values.

The textural properties of each sample were further analyzed to clarify the variation in the pore structure by thermal annealing and controlling the oxidation degree of GO (Table 1, Table S2). The specific surface area of ArGO increased noticeably from 30.8 m^2^/g of GO to 436.0 m^2^/g after the first rapid thermal annealing, and the specific surface area of TArGO further increased to 653.2 m^2^/g. When the specific surface area was divided into micropores and external pores, the surface area of the micropores increased from 115.2 to 367.2 m^2^/g, whereas that of external pores decreased from 320.8 to 286 m^2^/g after the second thermal annealing in N_2_. This tendency was also observed in the calculation of pore volume, showing increased total and micropore volume but decreased external pore volume after the second thermal annealing. This observation indicates that the second thermal annealing is effective in increasing the specific surface area by generating additional micropores, possibly due to the facilitated etching of carbon and oxygen from the basal plane of ArGO^37^. The surface values of TArGO surpassed those of the commercial rGO powder (V20-rGO) with values of 462 m^2^/g for the total surface area and 175.2 m^2^/g for the micro-surface area (Fig. S4), indicating the effectiveness of our approach for preparing highly porous graphene materials. In contrast, the decreasing external surface area of TArGO (286 m^2^/g) over that of ArGO (320.8 m^2^/g) is attributable to the slightly enhanced stacking between the rGO nanosheets, as evidenced by the SEM image and XRD patterns of TArGO indicated above. In addition, we measured the rGO annealed in N_2_ at 600 °C for 2 h without rapid thermal annealing in the air (Fig. S5). The graphene sample showed a specific surface area of 635.6 m^2^/g, which is similar to that of TArGO. However, the micro-surface area was significantly decreased to 51.4 m^2^/g, indicating that rapid annealing in the air is critical for preparing nanoporous graphene with a dense micropore.

**Table 1:** Textural properties of prepared materials, including surface areas and pore volumes.

Sample	S_BET_^a^ (m^2^/g)	S_external_ (m^2^/g)	S_micro_^b^ (m^2^/g)	V_total_^c^ (cm^3^/g)	V_external_ (cm^3^/g)	V_micro_^d^ (cm^3^/g)
Graphite	6.73	5.78	0.95	0.018	0.016	0.002
GO	30.8	5.3	25.5	0.03	0.01	0.02
V20-rGO^e^	462.0	286.8	175.2	0.96	0.75	0.21
rGO (N_2_ annealed)	635.6	584.2	51.4	1.74	1.59	0.15
ArGO	436.0	320.8	115.2	0.9	0.76	0.14
TArGO	653.2	286	367.2	1.08	0.70	0.38

^a^Brunauer–Emmett–Teller surface area calculated in the pressure range (P/P_0_) of 0.01–0.12.

^b^Micropore surface areas calculated from the N_2_ adsorption isotherms using the t-plot method.

^c^Total pore volume obtained at 0.99 of P/P_0_.

^d^Micropore volume calculated using the t-plot method.

^e^Commercial reduced graphene oxide (rGO) powder purchased from Standard Graphene.

In addition, the numbers of micropores and external pores can be controlled by adjusting the oxidation degree of GO, which is simply achievable by adjusting the oxidation time (Figs. S3, S6; Tables S2, S3). While ArGO samples prepared from GO with different oxidation degrees showed identical type-4 isotherms of N_2_ adsorption with similar Brunauer–Emmett–Teller surface areas of 430–470 m^2^/g, the numbers of micropores of ArGO increased significantly when the GO samples with a low degree of oxidation were used for a rapid thermal treatment. In contrast, a larger external surface area was measured for GO samples having higher oxidation degrees (Table S2), because the expansion of the graphene sheet can be facilitated by the decomposition of more oxygen groups into CO_2_ and CO. Interestingly, when ArGO was treated by 2nd annealing in nitrogen, the both micro and external surface area increased regardless of the oxidation degrees of staring GO (Table S3). Considering the aforementioned phenomena, we conclude that the hierarchical pore structure of graphene can be easily tuned by controlling the oxidation degree of GO or by conducting second thermal annealing under inert conditions.

**Figure 2:**
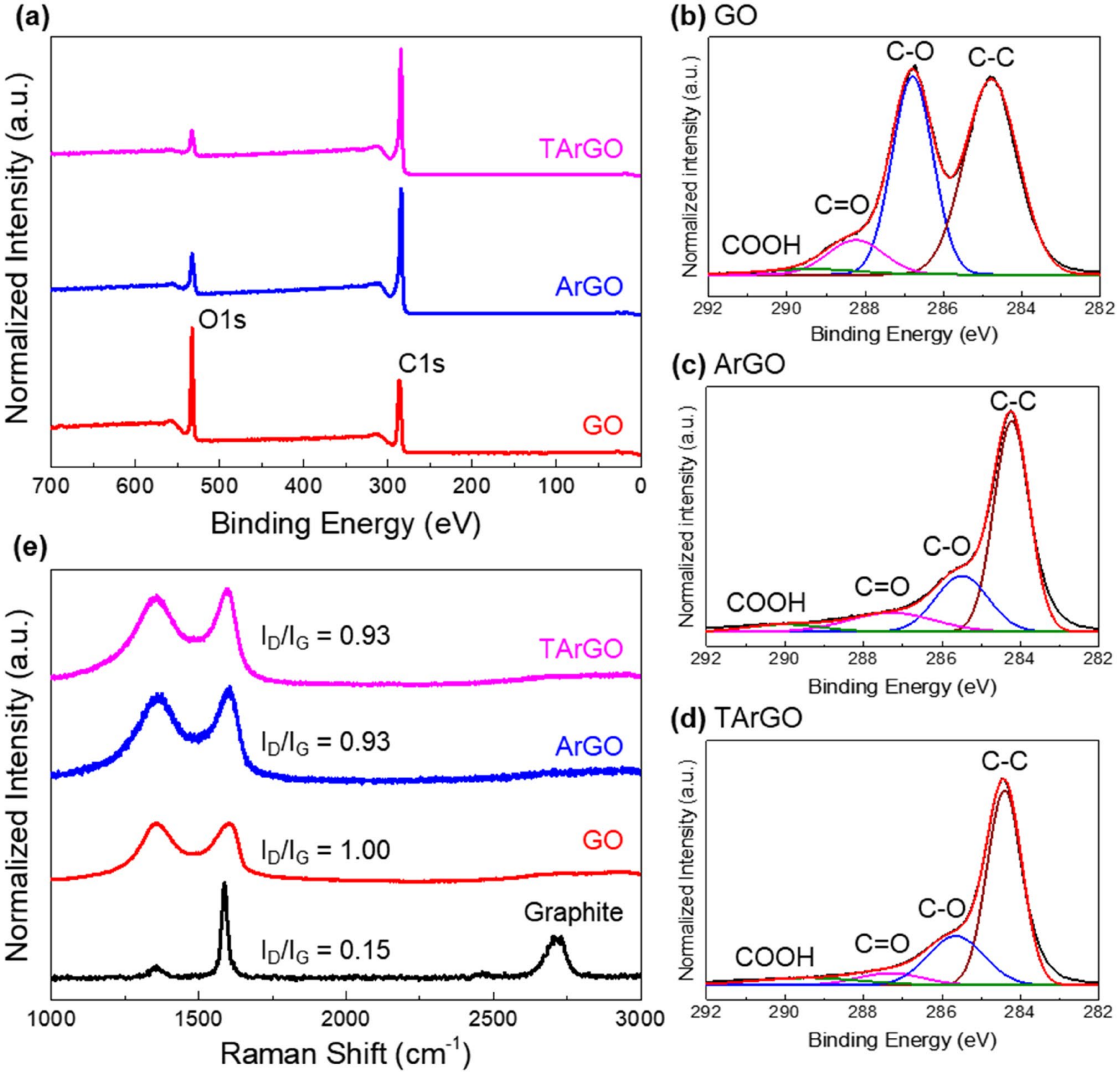
Chemical properties of Graphene oxide (GO), activated reduced GO (ArGO), and thermally treated ArGO (TArGO). (**a**) X-ray photoelectron spectroscopy survey scan of GO, ArGO, and TArGO. (**b–d**) XPS C 1 s spectra of GO, ArGO, and TArGO, respectively. (**e**) Raman spectra of graphite, GO, ArGO, and TArGO.

To investigate the chemical properties of ArGO and TArGO, characterization was conducted using XPS and Raman spectroscopy (Fig. 2). The XPS survey scan spectra demonstrated the presence of carbon and oxygen in GO, ArGO, and TArGO in Fig. 2a, where the number of oxygen groups differed for each sample. The amount of oxygen in GO (30 min of oxidation) was 29% and that in ArGO decreased to 12%, because most oxygen groups were removed by rapid thermal annealing. TArGO showed a further decrease in the peak intensity of oxygen to 8% because the residual oxygen groups were further reduced by the second annealing in the inert gas. The variation in the oxygen-containing groups was systematically investigated using the XPS C 1 s spectra, as shown in Fig. 2b–d. The GO formed after 30 min of oxidation was decorated with abundant oxygen-containing groups, such as C–O (286.78 eV), C=O (288.23 eV), and COOH (289.43 eV) (Fig. 2b). After the first thermal annealing, the number of overall oxygen-containing groups decreased, and the C–O group showed a significant reduction from 38 to 23%. The C–O group content reduced from 23 to 18% after second thermal annealing. In contrast, other oxygen-containing groups, such as C=O and COOH, were not completely removed even after the dual thermal treatment. This phenomenon can be explained by the thermal stability of oxygen-containing groups because the dissociation of OH groups begins to occur at a relatively low temperature of ~ 150 °C as compared to the other oxygen-containing groups^40^.

The Raman spectra were investigated to distinguish the crystal structure of the carbon materials in Fig. 2e. The intensity ratio of the D/G-band of ArGO and TArGO decreased from 1.0 of GO to 0.93 due to the decomposition of oxygen-containing groups. The oxidation degree of GO did not affect the intensity ratio of the D/G-band of ArGO (Fig. S7). The widths of the D and G bands of ArGO and TArGO were broader than those of GO, implying that the graphene sheets comprised amorphous and turbostratic carbon rather than sp^2^ carbon domains^41^, because severe defective structures were formed during the decomposition of oxygen-containing groups^42^ and 600 °C was too low a temperature to induce the crystallization of amorphous carbon into the graphitic crystal^38, 39^.

**Figure 3:**
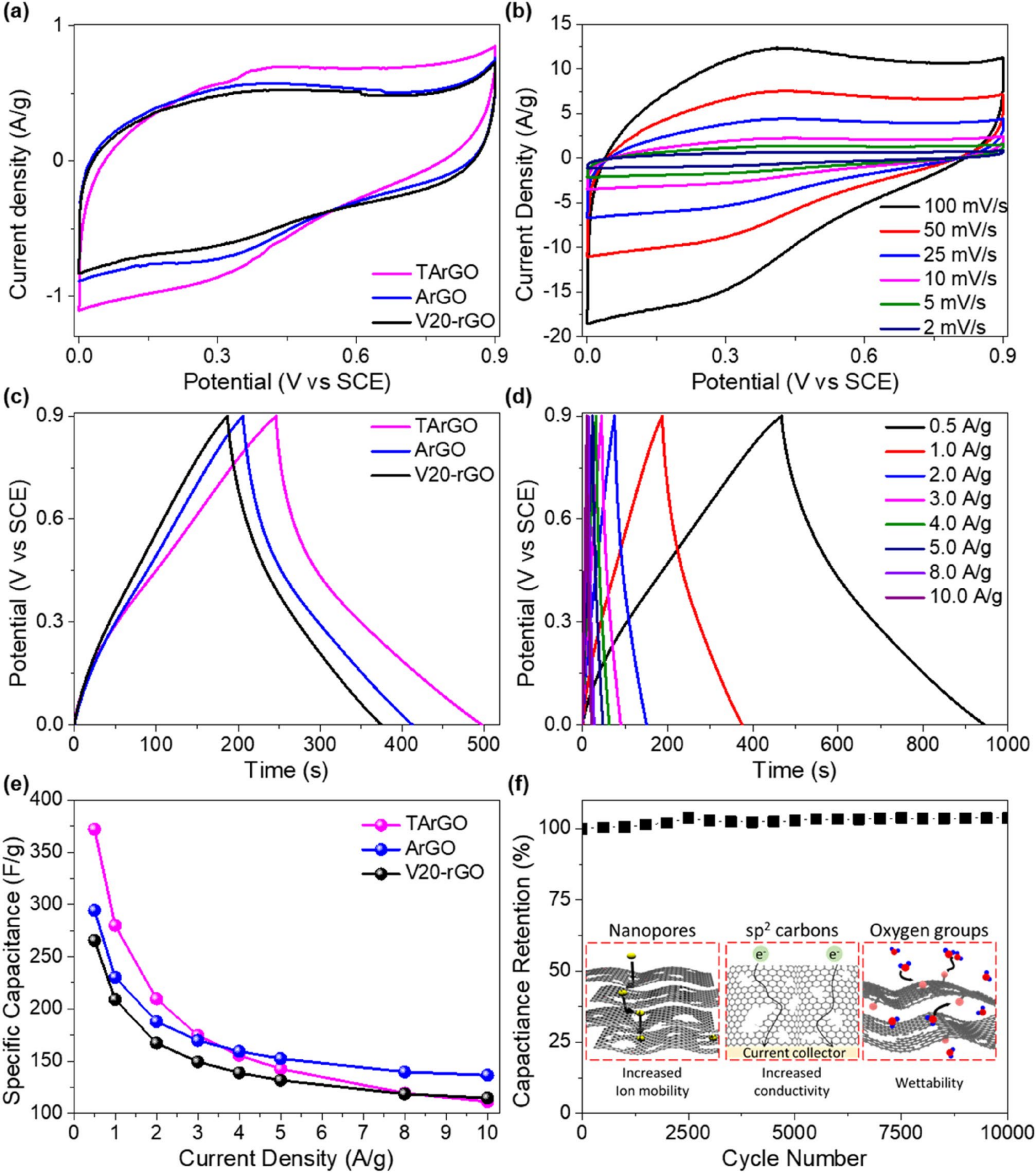
(**a**) Cyclic voltammetry curves of V20-rGO, activated reduced graphene oxide (ArGO), and thermally treated ArGO (TArGO) at a scanning rate of 2 mV/s. (**b**) Cyclic voltammetry curves of TArGO depending on the potential sweep rates. (**c**) Galvanostatic charge/discharge curves of V20-rGO, ArGO, and TArGO under a constant current density of 1 A/g. (**d**) Galvanostatic charge/discharge curve of TArGO depending on the constant current densities. (**e**) Specific capacitances of V20-rGO, ArGO, and TArGO as functions of constant current density. (**f**) Specific capacity vs cycle number of TArGO at a constant current density of 10 A/g.

To clarify the influence of the hierarchical pore structure of graphene on the supercapacitor performance, commercial rGO (V20-rGO), ArGO, and TArGO were tested as electrodes, as shown in Fig. 3 and Fig. S8. Figure 3a compares the specific capacitances of V20-rGO, ArGO, and TArGO at a scan rate of 2 mV/s within the potential range of 0.0–0.9 V. As expected from the largest surface area of TArGO (Table 1), the largest area under the CV curve was observed in comparison to V20-rGO and ArGO at the same scan rate. The CV plots of all samples showed a nearly rectangular shape, indicating that the main charge-storage process occurred through an electrical double-layer capacitance (EDLC) mechanism due to the large specific surface area of the carbon materials^43^. The presence of redox peaks was also observed near the 0.3–0.5 V region for all tested graphene samples, because the oxygen-containing groups, including C–O, C=O, and COOH, acted as redox-active sites^44^. The CV plots of TArGO were also investigated at different scan rates ranging from 2 to 100 mV/s, as shown in Fig. 3b, verifying the EDLC mechanism even at the high scan rates, while the area of the CV plots decreased as the Faradaic reaction could not be completed within the given time.

Figure 3c,d show the GCD curves of V20-rGO, ArGO, and TArGO at a constant current density of 1 A/g and that of TArGO at different current densities, respectively. The GCD curves of all graphene samples showed triangular shapes, reconfirming that the ion storage mainly occurred through EDLC, as observed in the CV plots shown in Fig. 3c. The discharge curves of all samples slightly deviated from linearity, which indicated an ohmic drop due to the occurrence of a pseudocapacitance in the presence of oxygen-containing groups^45^. For comparison, the CV curves at a scan rate of 2–100 mV/s for V20-rGO and ArGO, as well as the GCD curves at constant current densities of 0.5–10.0 A/g for V20-rGO and ArGO, are summarized in Fig. S8.

To verify the influence of the micro- and external surface areas of graphene on the specific capacitance, the specific capacitances of V20-rGO, ArGO, and TArGO were compared in a constant current density range of 0.5–10 A/g (Fig. 3e). As expected from the previous porous graphene and carbon materials^46, 47^, the specific capacitance of TArGO improved drastically over those of ArGO and V20-rGO for all current density values; in particular, TArGO exhibited excellent performance up to 372.1 F/g at 0.5 A/g. Interestingly, TArGO exhibited higher specific capacitance than ArGO in the current density range of 0.5–3 A/g. However, it showed a lower specific capacitance than ArGO above a current density of 4 A/g. The transition of specific capacitance between ArGO and TArGO can be attributed to their pore structures, as reflected by the surface area shown in Table 1. ArGO was composed of a larger external surface area (320.8 m^2^/g) than TArGO (286 m^2^/g), while TArGO was mainly composed of micropores with an extremely large micro-surface area of 367.2 m^2^/g, which originated during the second thermal annealing conducted under inert conditions; the micro-surface area was much larger than that of ArGO (115.2 m^2^/g). The total surface area with both external and micro-surfaces can be utilized for EDLC-type charge storage at lower current densities. Therefore, TArGO with a larger total surface area can exhibit better charge-storage performance at lower current densities. In contrast, during the fast-charging process at higher current densities, the micro-surface area became less effective and the charge-storage process mainly occurred on the external surface area and oxygen-containing groups (Fig. S9). Consequently, ArGO can exhibit higher performance at a high current density. Nonetheless, the specific capacitance of both ArGO and TArGO was better than that of the commercial rGO in the observed range of current density, which indicates the advantage of hierarchical graphene materials as supercapacitor electrodes.

The durability of TArGO was investigated by observing the cycling stability performance of the TArGO electrode over 10,000 charge and discharge cycles at 10 A/g (Fig. 3f). After 10,000 cycles, the capacitance retention was 103.8%, which indicates that TArGO exhibits excellent cyclic stability as a supercapacitor electrode. The specific capacitance of TArGO increased because some redox passive oxygen-containing groups became redox-active by extending the conjugation during the cycle test of charge and discharge^44^.

The electrochemical performances of ArGO and TArGO were compared with those of previous carbon-based materials, as shown in Table 2^11, 44, 48–56^. The specific capacitance of TArGO (373.1 F/g at 0.5 mV/s and 280.0 F/g at 1 mV/s) was stable and higher than that of previous carbon materials, which can be attributed to the hierarchical nanoporous graphene structure with partial oxygen groups, as summarized in the inset of Fig. 3f. Hierarchical nanoporous structures with high surface areas facilitate ion transport between the graphene surface and electrolyte solution. A high-temperature treatment is also effective in enhancing the electrical conductivity of graphene materials, as reflected by the fact that the conductivity of TArGO (55.7 S/m) was slightly higher than those of ArGO (50.9 S/m) and commercial rGO (43.1 S/m) (Fig. S10), which is beneficial for providing a conducting pathway to the current collector. In addition, the residual oxygen-containing groups of TArGO and ArGO improve the wettability between the graphene surface and aqueous electrolyte, which was 1 M H_2_SO_4_ in this experiment, and increase the capacitance by additional pseudocapacitance.

**Table 2:** Comparison of electrochemical performance of supercapacitors based on carbon and graphene materials.

Electrode material	Method	Electrolyte	Current density (A/g)	ΔV	Cs (F/g)	Cycle test	References
Activated carbon fiber	Chemical activation	1 M H_2_SO_4_	0.2	1.8	254.0	10,000 (96%)	^48^
GO/Ppy	Polymerization	1 M H_2_SO_4_	1.0	1.0	233.0	4000 (91.2%)	^49^
GTR	Surfactant intercalation	2 M H_2_SO_4_	1.0	1.0	194.0	1000 (90%)	^50^
RGO	Hydrothermal treatment	1 M H_2_SO_4_	1.0	0.8	367.0	1000 (107.7%)	^44^
RGO-HD	Thermal annealing	6 M KOH	1.0	1.0	182.0	5000 (96.4%)	^51^
RGO-HP5A	Redox reaction	1 M H_2_SO_4_	1.0	1.0	294.0	–	^52^
rlGO	Hydrothermal treatment	1 M H_2_SO_4_	1.0	1.0	274.0	–	^53^
T-GR	Thermal annealing	6 M KOH	1.0	1.0	271.0	5000 (93.8%)	^54^
G250	Thermal annealing	6 M KOH	1.0	1.0	167.3	3000 (94.7%)	^55^
P-3DHPCs	Pyrolysis	6 M KOH	0.3	1.0	319	10,000 (96.5%)	^11^
ArGO	Thermal annealing	1 M H_2_SO_4_	1.0	0.9	230.3	–	This work
TArGO	Thermal annealing	1 M H_2_SO_4_	0.5	0.9	372.1	–	This work
			1.0	0.9	280.0	10,000 (103.8%)	This work

## Conclusions

We demonstrated a simple yet effective method for preparing hierarchical nanoporous graphene using a dual thermal activation approach. First, rapid thermal annealing was introduced to generate a hierarchical pore structure by decomposing the oxygen-containing groups of GO. Then, the second thermal annealing was performed under N_2_ conditions to generate dense micropores on the basal plane of graphene while maintaining the hierarchical structure. A supercapacitor composed of TArGO showed a specific capacitance up to 372.1 F/g at 0.5 A/g and 280.0 F/g at 1 A/g in 1 M H_2_SO_4_ with excellent long-term stability even after 10,000 cycle tests. The excellent capacitance of TArGO can be attributed to EDLC, in addition to the slight contribution of the pseudocapacitance by the oxygen-containing groups. The suggested approach is not only effective for controlling the pore structure of graphene but is also highly scalable because of its simple process in the absence of additional chemicals.

## Supplementary Information

Supplementary Information
