# Author Correction: Tuning the hierarchical pore structure of graphene oxide through dual thermal activation for high-performance supercapacitor

**DOI:** 10.1038/s41598-021-87519-x

**Published:** 2021-04-08

**Authors:** Jeongpil Kim, Jeong-Hyun Eum, Junhyeok Kang, Ohchan Kwon, Hansung Kim, Dae Woo Kim

**Affiliations:** Department of Chemical and Biomolecular Engineering, Yonsei University, Yonsei-ro 50, Seodaemun-gu, Seoul, 120-749 Republic of Korea

Correction to: *Scientific Reports* https://doi.org/10.1038/s41598-021-81759-7, published online 22 January 2021

In the original version of this Article, the Acknowledgements section was incomplete.

“This work was supported by the Basic Science Research Program through the National Research Foundation of Korea funded by the Ministry of Education (NRF-2019R1A6A1A1105566012).”

now reads:

“This work was supported by the Basic Science Research Program through the National Research Foundation of Korea funded by the Ministry of Education (NRF-2019R1A6A1A1105566012). This work was supported by the National Research Foundation of Korea (NRF) grant funded by the Korea government (MSIT) (NRF-2020R1C1C1003289). This work was supported by the Technology Innovation Program (20013621, Center for Super Critical Material Industrial Technology) funded By the Ministry of Trade, Industry & Energy (MOTIE, Korea).”

The original Article has been corrected.

