# Supplementary material for: Tuning the hierarchical pore structure of graphene oxide through dual thermal activation for high-performance supercapacitor
Source: Sci Rep. 2021 Jan 22;11:2063. doi: 10.1038/s41598-021-81759-7 (PMC7822934; doi:10.1038/s41598-021-81759-7)
Supplement: Supplementary file 1 — Supplementary Information [file 41598_2021_81759_MOESM1_ESM.docx]

Supplementary Material for

**Tuning the Hierarchical Pore Structure of Graphene Oxide through Dual Thermal Activation for High-Performance Supercapacitor**

*Jeongpil Kim*^†^*, Jeong-Hyun Eum*^†^*, Junhyeok Kang, Ohchan Kwon, Hansung Kim*, Dae Woo Kim**

Department of Chemical and Biomolecular Engineering, Yonsei University, Yonsei-ro 50, Seodaemun-gu, Seoul 120-749, Republic of Korea

*Emails: elchem@yonsei.ac.kr (H. Kim) and audw1105@yonsei.ac.kr (D.W. Kim)

**Table S1**. Experimental conditions used for preparing GO with different oxidation degrees.


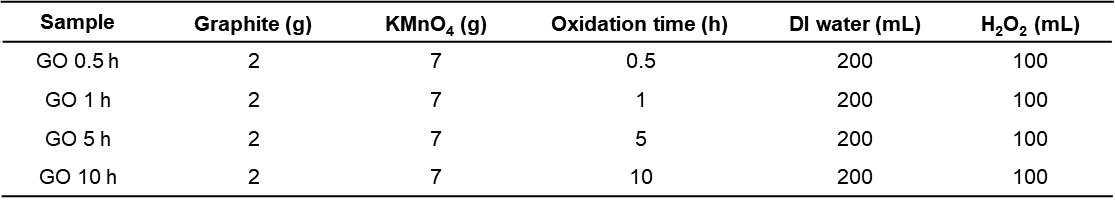


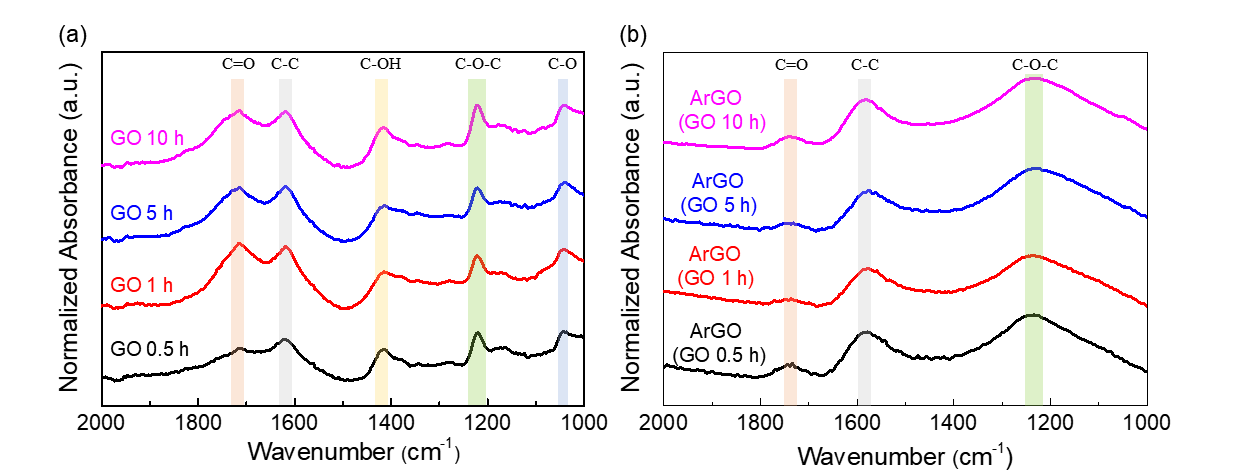


**Fig. S1.** Fourier transform infrared spectra of **(a)** graphene oxide and **(b)** activated reduced graphene oxide with different oxidation times. The ratio of graphite and KMnO_4_ was fixed at 3.5 and the oxidation time was varied from 30 min to 10 h.


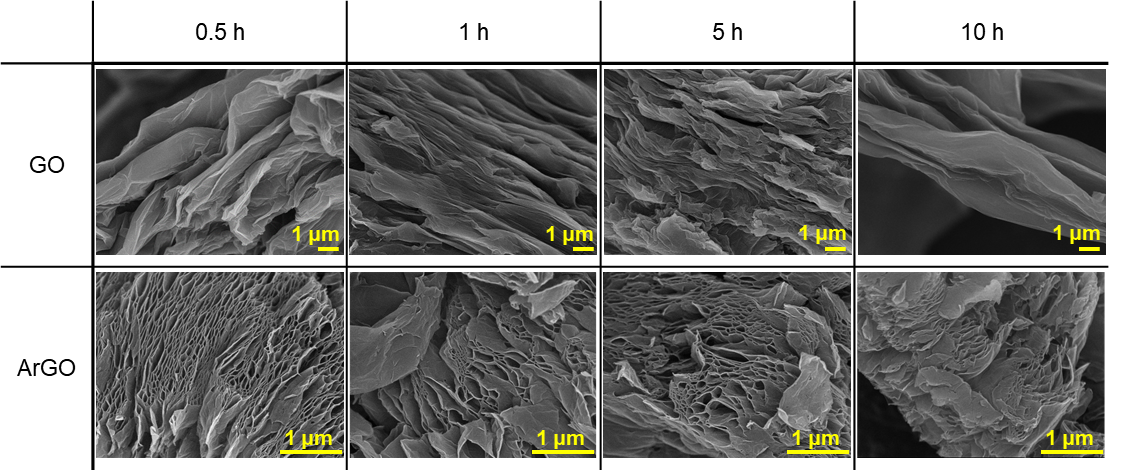


**Fig. S2.** SEM images of graphene oxide (GO) and activated reduced graphene oxide (ArGO) depending on the oxidation time of GO. The slit nanopore structure of ArGO was successfully generated regardless of the oxidation degree of GO.


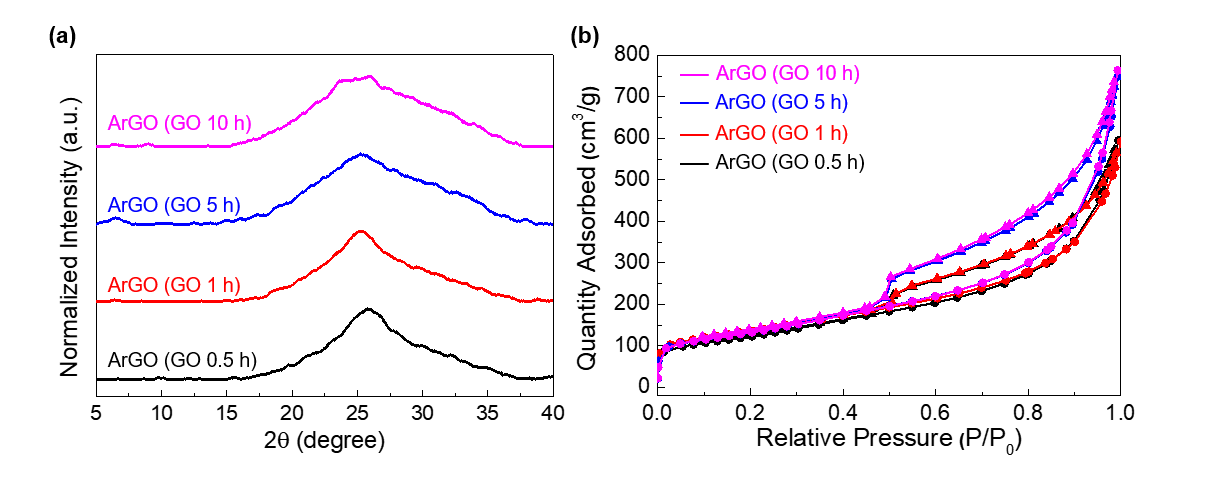


**Fig. S3.** **(a)** XRD patterns of activated reduced graphene oxide (ArGO) with different oxidation times of graphene oxide (GO). **(b)** N_2_ adsorption-desorption isotherms of ArGO obtained at 77 K depending on the oxidation time of GO.

**Table S2.** Textural properties of the prepared materials, including surface areas and pore volumes.


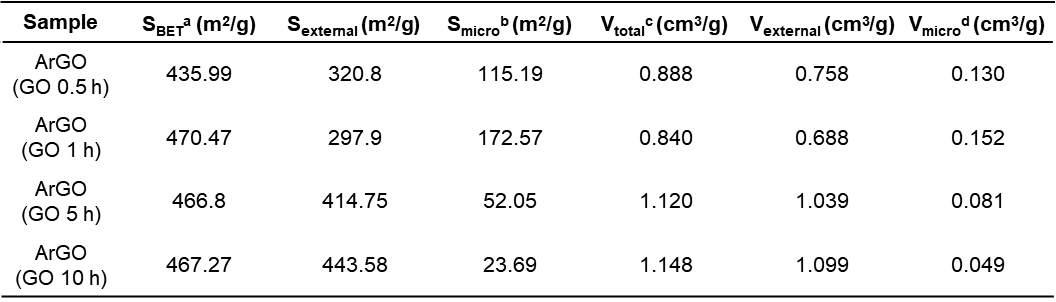


**^a^** Brunauer–Emmett–Teller surface area calculated in the pressure range (P/P_0_) of 0.01−0.12.

**^b^** Micropore surface areas calculated from the N_2_ adsorption isotherms using the t-plot method.

**^c^** Total pore volume obtained at 0.99 of P/P_0_.

**^d^** Micropore volume calculated using the t-plot method.

The external pore volume and surface area were obtained by subtracting the micropore volume and micropore surface area from the total values.


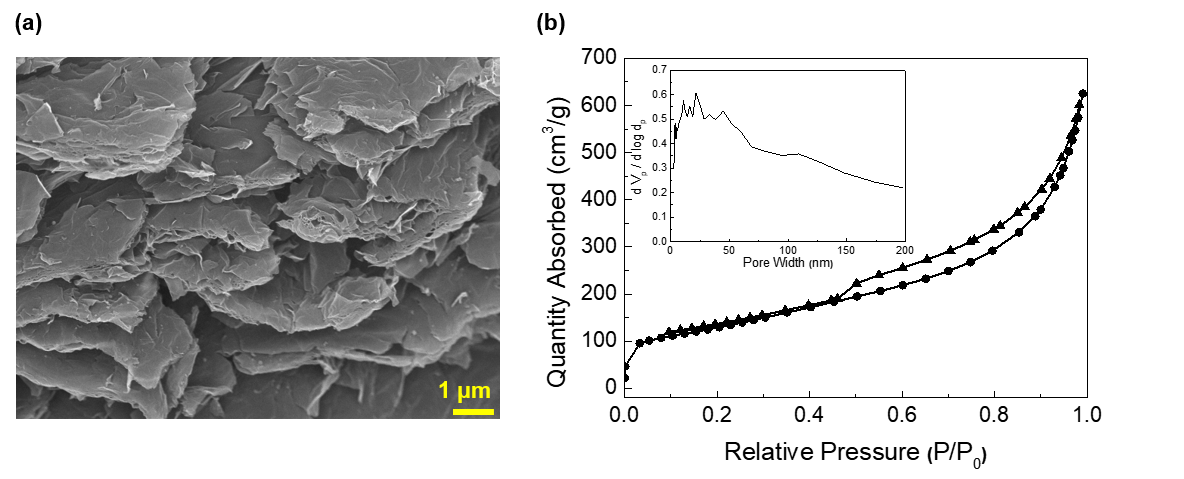


**Fig. S4.** **(a)** SEM image of commercial reduced graphene oxide (V20-rGO) powder purchased from standard graphene. **(b)** N_2_ adsorption-desorption isotherms of V20-rGO at 77 K. The inset shows a pore size distribution of V20-rGO calculated using the BJH method.


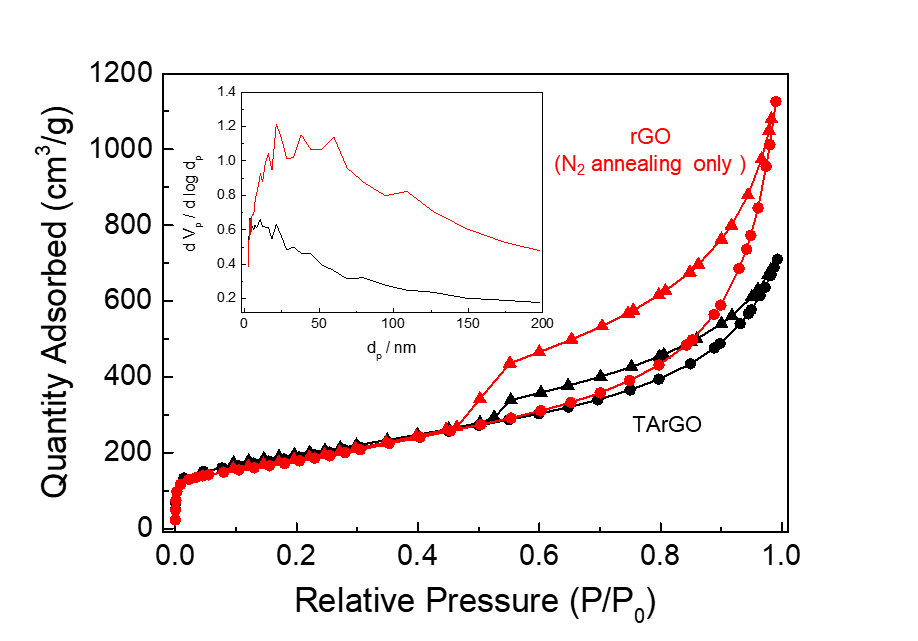


**Fig. S5.** N_2_ adsorption-desorption isotherms of rGO annealed in N_2_ at 600˚C for 2 h without rapid thermal annealing in the air in comparison with TArGO. The isotherms were obtained at 77 K. Inset is pore size distributions calculated by the BJH method.


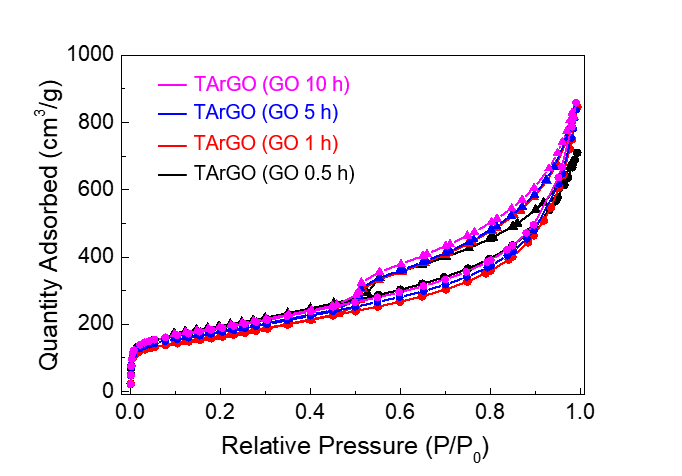


**Fig. S6.** N_2_ adsorption-desorption isotherms of TArGO obtained at 77 K depending on the oxidation time of GO.

**Table S3.** Textural properties of TArGO depending on the oxidation time of GO.


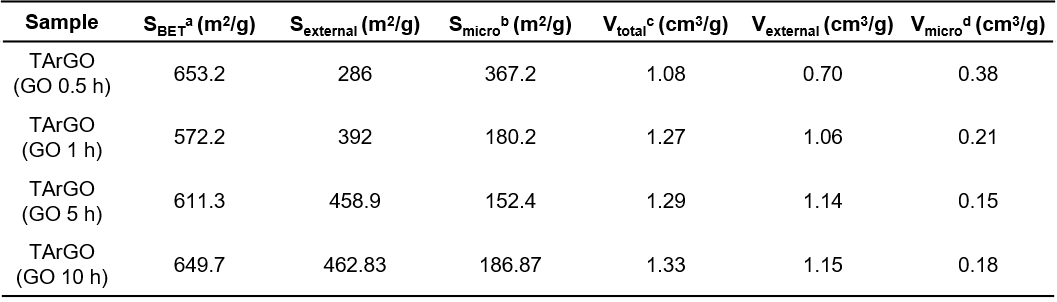


**^a^** Brunauer–Emmett–Teller surface area calculated in the pressure range (P/P_0_) of 0.01−0.12.

**^b^** Micropore surface areas calculated from the N_2_ adsorption isotherms using the t-plot method.

**^c^** Total pore volume obtained at 0.99 of P/P_0_.

**^d^** Micropore volume calculated using the t-plot method.

The external pore volume and surface area were obtained by subtracting the micropore volume and micropore surface area from the total values.


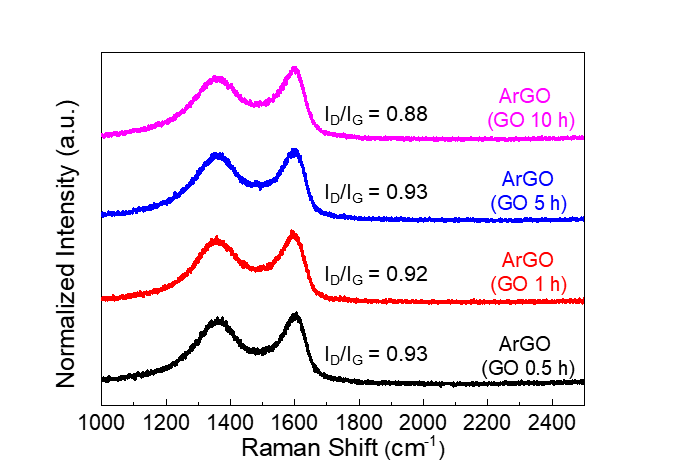


**Fig. S7.** Raman spectra of activated reduced graphene oxide with different oxidation times. The ratio of graphite and KMnO_4_ was fixed at 3.5 and the oxidation time of graphene oxide was varied from 30 min to 10 h. A drastic change in peak shape was not observed and all samples showed a typical characteristic of amorphous carbon bonding with broad D and G-band peaks.


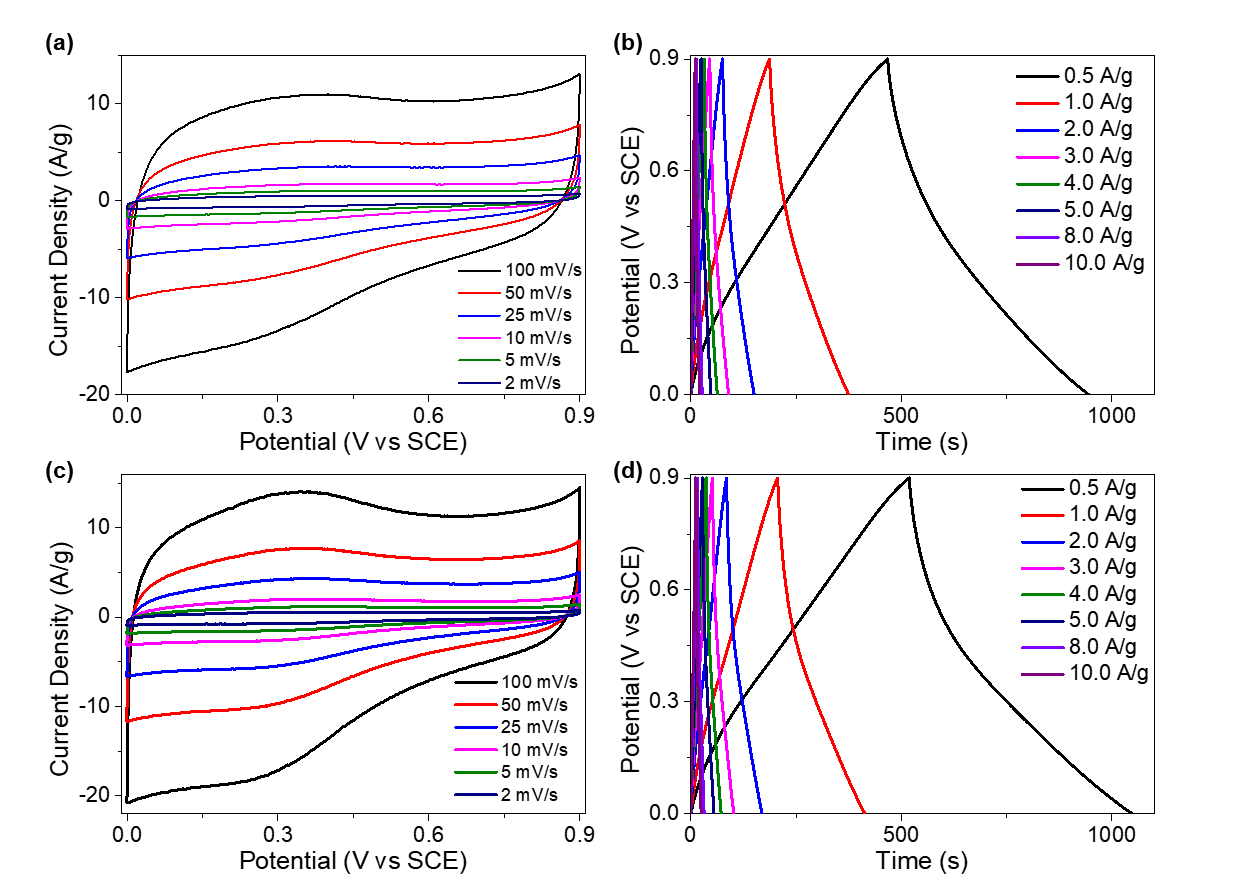


**Fig. S8.** **(a)** Cyclic voltammetry curve of commercial reduced graphene oxide (V20-rGO) with different potential sweep rates. **(b)** Galvanostatic charge/discharge curves of V20-rGO under different constant current densities. **(c)** Cyclic voltammetry curve of activated reduced graphene oxide (ArGO) with different potential sweep rates. **(d)** Galvanostatic charge/discharge curves of ArGO under different constant current densities.


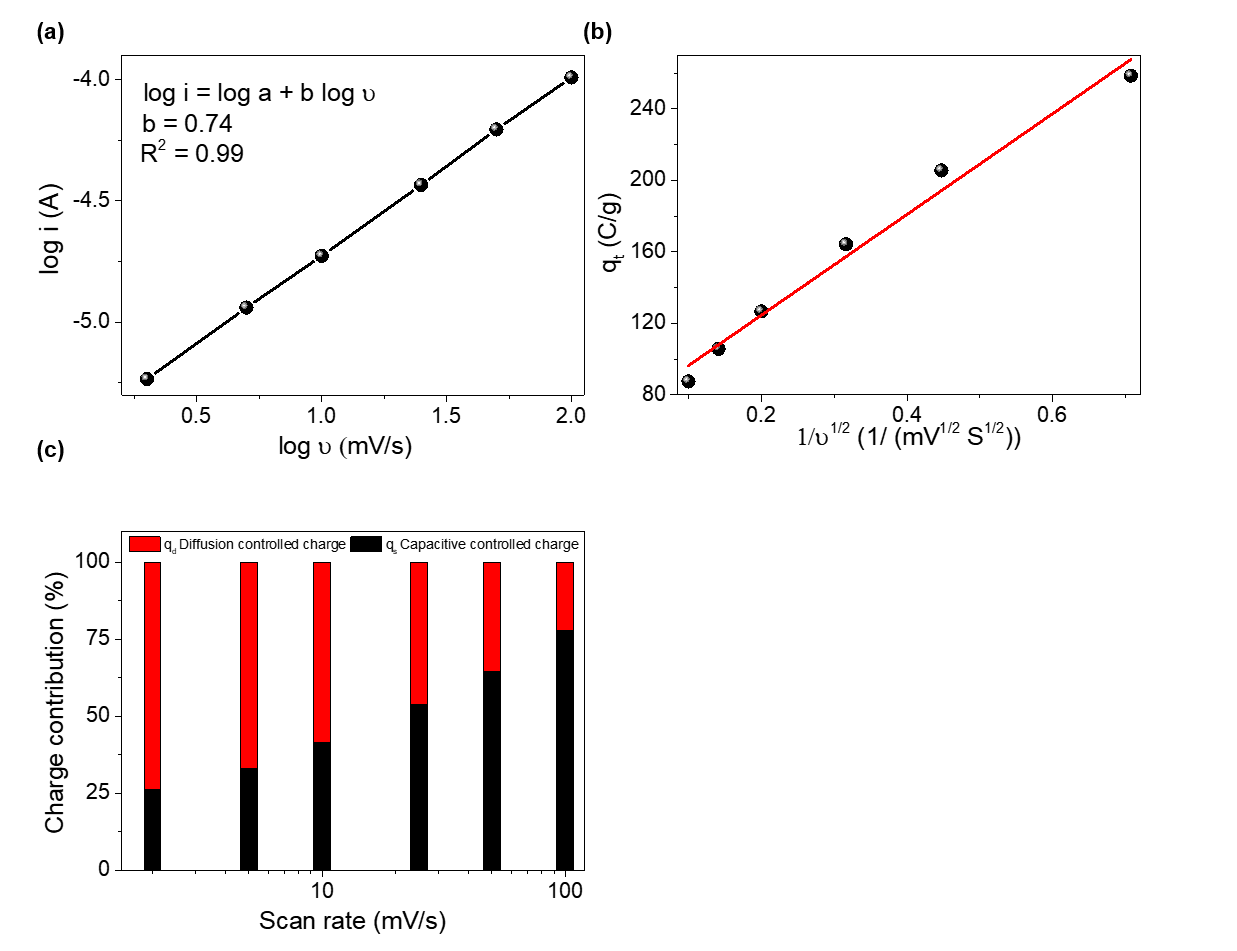


**Fig. S9.** **Kinetic study of charge storage of TArGO.** **(a)** Peak current as a function of the scan rate (ν). **(b)** Total charge as a function of 1/ ν^1/2^. **(c)** Capacitive and diffusion contributions to the total charge storage in the scan rate range from 2 to 100 mV/s.

The charge storage kinetics of TArGO electrodes were investigated from CV plots using the following power-law relationship (**equation 1**).

$\boldsymbol{i=a}\boldsymbol{\upsilon}^{\boldsymbol{b}}$ **(1)**

Here, $i$ is the current for a particular voltage at scan rate $\upsilon$, a and b are the coefficients. The nature of charge storage can be estimated based on the value of b, which is the slope of the log $i$ vs log $\upsilon$ plot. b = 1 indicates a surface-capacitive charge storage process, whereas b = 0.5 indicates a diffusion-controlled charge-storage process. In log $i$ vs log $\upsilon$ plot for anodic peak current potential (**Fig. S9a**), the value of b is 0.74, indicating that performance of TArGO was achieved by the contribution of both surface capacitive and diffusion controlled charge-storage process.

In the CV curve, the total charge storage (q_t_) was contributed by the surface capacitive (q_s_) and diffusion controlled (q_d_) processes as shown in **equation 2**.

So, $\boldsymbol{q}_{\boldsymbol{t}}\boldsymbol{=}\boldsymbol{q}_{\boldsymbol{s}}\boldsymbol{+}\boldsymbol{q}_{\boldsymbol{d}}$ **(2)**

Or, $\boldsymbol{q}_{\boldsymbol{t}}\boldsymbol{=}\boldsymbol{q}_{\boldsymbol{s}}\boldsymbol{+c}\boldsymbol{v}^{\boldsymbol{-1/2}}$ **(3)**

c is the constant and $v$ is the scan rate. **Fig. S9b** shows the total charge as a function of the reciprocal of the square root of the scan rate by considering a semi-infinite linear diffusion as shown in **equation 3**. From this plot $\boldsymbol{q}_{\boldsymbol{s}}$ value can be derived for $v$ = ∞. **Fig. S9c** shows the calculated contribution of q_s_ and q_d_ at each scan rate. The capacitive charge contribution increases with scan rate and reaches 78% at 100 mV/s, indicating that charge storage mainly occurred by a capacitive process of oxygen-containing groups at a high scan rate.


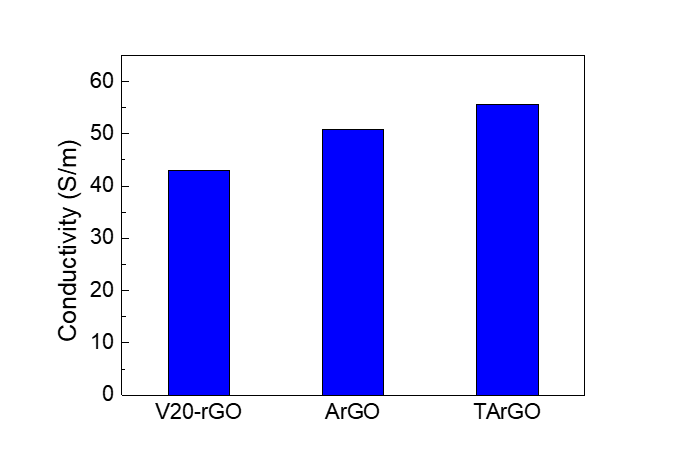


**Fig. S10.** The electrical conductivity of commercial reduced graphene oxide (V20-rGO), activated reduced graphene oxide (ArGO), and thermally treated ArGO (TArGO). All samples were prepared in the form of a pellet of dimensions 1 cm × 3.6 cm. The pellets were formed by hot-pressing graphene powders at 25 ℃ with a pressure of 2.6 ton/cm^2^ for 4 min. The sheet resistance of the pellet was measured using a four-probe instrument and converted to electrical conductivity. The electrical conductivity of graphene was slightly enhanced after the dual annealing process.
